# The impact of community health workers (CHWs) on Buruli ulcer in sub-Saharan Africa: a systematic review

**DOI:** 10.11604/pamj.2013.15.19.1991

**Published:** 2013-05-10

**Authors:** Marius Zambou Vouking, Violette Claire Tamo, Lawrence Mbuagbaw

**Affiliations:** 1 Center for the Development Best Practices in Health, Yaoundé Central Hospital, Henri-Dunant Avenue, Messa, Yaoundé, Cameroon; 2 Department of Clinical Epidemiology and Biostatistics, McMaster University, Hamilton, ON, Canada

**Keywords:** Systematic review, impact, Community Health Workers, Buruli ulcer, sub-Saharan Africa

## Abstract

Buruli ulcer (BU) is a cutaneous neglected tropical disease caused by Mycobacterium ulcerans. Participation of Community Health Workers (CHWs) is an integral part of the management of BU, yet their impact has not been systematically evaluated in sub-Saharan Africa. METHODS: Our objectives were to summarize the evidence on the impact of CHWs on the control of BU in sub-Saharan Africa by looking at their recruitment, training, non-governmental support and performance. We searched the following electronic databases from January 1998 to July 2012: Medline, EMBASE (Excerpta Medica Database), The Cochrane Library, Google Scholar, CINAHL (Cumulative Index to Nursing and Allied Health Literature), WHOLIS (World Health Organization Library Database), LILACS (Latin American and Caribbean Literature on Health Sciences) and contacted experts in the field. There were no restrictions to language or publication status. All study designs that could provide the information we sought were eligible, provided the studies were conducted in sub-Saharan Africa. Critical appraisal of all identified citations was done independently by two authors to establish the possible relevance of the articles for inclusion in the review. Of 195 hits, 17 papers met the inclusion criteria. For the management of Buruli Ulcer, CHWs are often recruited from the communities they will serve. Communities play a role in CHW selection. Larger numbers of CHWs are needed in order to improve the detection and management of cases. One of the major obstacles to the control of BU is inadequate and poorly- equipped health facilities in the affected areas. Evidence from this review suggests that CHW programmes can have large impacts on the control of BU in sub-Saharan Africa. Large-scale rigorous studies, including RCTs, are needed to assess whether the CHWs programs promote equity and access.

## Introduction

Buruli ulcer (BU) is a cutaneous Neglected Tropical Disease (NTD) caused by *Mycobacterium ulcerans* [1, 2]. It is the third most frequent mycobacterial infection after tuberculosis and leprosy [1, 2]. It is characterized initially by a nodule that later progresses to vast cutaneous ulcerations, mediated by Mycolactone, a toxin secreted by *M. ulcerans* [3, 4]. The exact mode of transmission is the subject of research [5]. BU is endemic in thirty countries, and is suspected to exist in 10 other countries of the African Region [1]. The World Health Organization (WHO) initiated the Global Buruli Ulcer Initiative (GBUI), in response to the growing spread of BU [6], and since 1998 the GBUI has been supporting research and improving the management of BU.

In developing countries, the health systems are weakened by human resources shortages. Therefore, evidence-based interventions may not be implemented as required [7–9]. Community Health Workers (CHWs) are essential components of the healthcare workforce who do not require the lengthy and costly training of health personnel and can have a considerable impact in health care [8, 9].

The WHO describes CHWs as follows: “(they) should be members of the communities where they work, should be selected by the communities, should be answerable to the communities for their activities, should be supported by the health system but not necessarily a part of its organization, and have shorter training than professional workers” [9]. CHWs find themselves in the best position to deliver services in well mobilised communities. They work in hand-in-hand with other health workers to provide primary health care (PHC) [1, 10, 11]. This is particularly true for poor, rural communities for whom the provision of preventive and curative services is the main entry point into the health system [1, 2, 8].

CHWs, by virtue of their proximity with the communities are able to play an important role in the detection and referral of cases of BU [2, 4, 6, 12]. In fact, they are members of the community and have equally high stakes in the health of the community. This can lead to timely management and therefore reduce the morbidity and mortality suffered by those infected with BU [12]. Lehmann et al. [13] and Lewin et al. [14] reviewed the evidence on CHW interventions in low- and middle-income countries (LMIC). They found lay health workers to be effective in specific areas of child health, when compared to usual care - community members fending for themselves [14]. Haines et al. [15] highlighted the context specific nature of a CHW's performance.

Synthesizing the evidence on their impact in the management of BU can help to better define their roles, identify weaknesses in how their activities are implemented and inform health systems on relevant measures that can be used to control BU. We therefore conducted a systematic review to summarize the evidence of the impact of CHWs on the control of BU in sub-Saharan Africa.

Our objectives were to summarize the evidence on the impact of CHWs in the control of BU in sub-Saharan Africa. The specific objectives of the studies were to describe CHWs in the control of BU in sub-Saharan Africa with special emphasis on their recruitment, training and involvement of non-governmental association; to describe the impact of CHWs on BU in term of the number of cases identified, referred and confirmed.

## Methods

**Search strategy:** We searched the following electronic databases from January 1998 to July 2012: Medline, EMBASE (Excerpta Medica Database), The Cochrane Library, Google Scholar, CINAHL (Cumulative Index to Nursing and Allied Health Literature), WHOLIS (World Health Organization Library Database), LILACS (Latin American and Caribbean Literature on Health Sciences) and contacted four experts in the field. The following search strategy was modified for the various databases and search engines: («Impact» OR «Contribution» AND «Community Health Worker» OR «Lay Health Worker» AND «*Mycobaterium ulcerans*» OR «Buruli ulcer» AND «Sub Saharan Africa» OR «Endemic country»). Along with MeSH terms and relevant keywords, we used the Cochrane Highly Sensitive Search Strategy for identifying reports of articles in Pubmed. There were no restrictions to language or publication status. Our search was limited to the last fourteen years, as they correspond to the period of enactment of the GBUI [6]. Prior to the GBUI, efforts to control BU, especially with CHWs were almost inexistent.

**Study design:** All study designs were eligible for inclusion provided they were on CHWs working on BU in sub-Saharan Africa.

**Study participants:** Owing to the wide range of functions that fall under the umbrella term “community health worker” we designed a definition of our own. For this review, we defined CHWs as lay individuals trained in the particular role of delivering curative or preventive care in the control of BU.

**Types of interventions:** We included interventions if the description was adequate for us to establish that it was a CHW intervention aimed at the control of BU. Where such details were unclear, we contacted the study authors, whenever possible, to establish whether the personnel described were CHWs. Four study authors were contacted.

>b>Data sought Any of the following data were sought: Type of training; Recruitment; Involvement of NGOs; Impact, defined as: number of cases identified, number of case referred and number of referred cases confirmed.

**Data extraction and management:** Critical appraisal of all identified citations was done independently by two authors (MZV and VCT) to establish the possible relevance of the articles for inclusion in the review. Studies were reviewed for relevance based on types of participants (CHWs), interventions (control of BU), and outcome measures. We retrieved full text copies of the articles identified as potentially relevant by either one or both review authors. Where appropriate, we contacted study authors for further information and clarification. Disagreements were resolved by consensus or by arbitration of a third review author (LM). The flow of study selection is described in a Preferred Reporting Items for Systematic Reviews and Meta-Analyses (PRISMA) diagram [16]. Data are reported in a narrative manner.

**Assessment of quality in included studies:** The included studies were not scored for quality.

### Current status of knowledge

**Figure 1:**
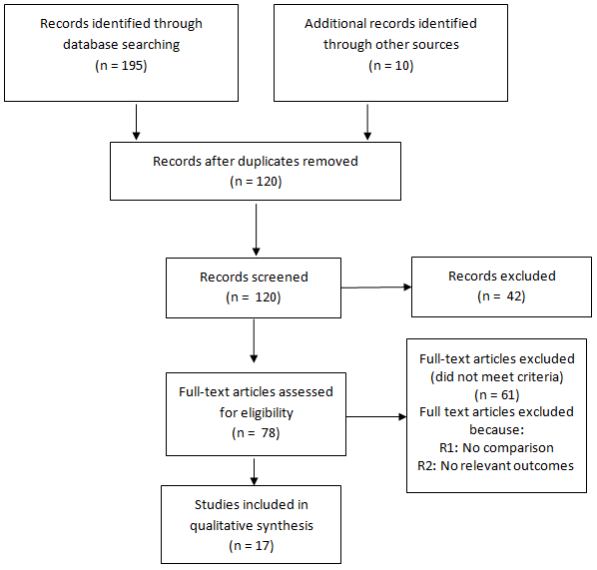
PRISMA flow diagram

Our searches retrieved 195 studies, of which 17 are included in the review (Figure 1, Table 1).

**Table 1:** Characteristics of included studies, CHWs performance and training; and NGO affiliation

	Reference	Type of study	Country of study	Recruitment of CHWs	Training	NGO affiliation	Impact measures
1	Nsom Mba, 2006 [26]	cross-sectional	Cameroon	By the community	Training of CHWS (general training)	None	Not reported
2	Um Boock, 2008 [34]	Case-control	Cameroon	By the community	Training of CHWS (general training)	Fairmed Foundation	Not reported
3	Um Boock et Deffo 2004 [35]	Case-control	Cameroon	Not reported	Training of CHWS (general training)	Fairmed Foundation	Not reported
4	Comte, 2006 [17]	Case-control	Cameroon	Village health committees	Not reported	“Médecins Sans Frontières”	Not Reported
5	China et Sopoh 2006 [22]	cross-sectional	Benin	By the community	Not reported	Raoul Foullereau.	Not reported
6	Sopoh et al. 2007 [23]	Cohort study	Benin	By the community	Support in the implementation of CHWs activities	None	Not reported
7	Malda, 2006 [18]	cross-sectional	Ivory Coast And Benin	Village health committees	Not reported	ANESVAD	Not reported
8	Ake, 2006 [19]	cross-sectional	Ivory Coast	By the community	Not reported	MAP International	Referral rate : 67%
9	Saunderson, 2006 [25]	cross-sectional	Ghana	By the community	Not reported	American Leprosy Missions	Not reported
10	Weeb et al. 2007 [12]	Randomed Controlled Trial	Ghana, Ivory coast	Village health committees	Not reported	None	Not reported
11	Adamba, 2011 [21]	controlled before and after	Ghana	Village health committees	Training of CHWs (general training)	None	Not reported
12	Kibadi et al. 2008 [24]	Cohort studies	Democratic republic of Congo	Not Reported	Training of CHWs in the early detection of cases	None	Not reported
13	Kibadi et al. 2009 [42]	cross-sectional studies	Democratic republic of Congo	By the community	Not reported	None	Not reported
14	Jonhson, 2005 [27]	cross-sectional studies	Benin	Not reported	Training of CHWs in case identification in	None	Not reported
15	Kanga et al. 2003 [30]	cross-sectional studies	Ivory Coast	By the community	Not reported	None	Referral rate : 65%
16	Vouking el al. 2010 [20]	cross-sectional studies	Cameroon	By the community	Training of CHWs in case identification	None	Referral rate : 95%
17	Ackumey et al. 2011 [41]	cross-sectional studies	Ghana	Not reported	Training of CHWs in case identification	None	Not reported

### Recruitment of CHWs

CHWs were nominated by village health committees or leaders in four studies [12, 17, 18, 21] and by community members in nine studies [19–26, 30, 34, 42]. Very little information was provided on their levels of education [20–24, 26].

### Training of CHWs

Here as well, many studies did not report on the training received by the CHWs. When it was reported, the training approaches varied greatly between studies and were not described on the same level of detail in all of them [20–27, 34, 35, 41]. The terms used included: courses, classes, seminars, workshops, discussion groups, practical training, and in-class practice [20–22, 24, 26, 27]. Facilitation was often done by national level facilitators who were from the Ministry of Health or NGOs [17–19, 22, 25, 34, 35].

### Role of NGOs

NGOs often initiated training activities [34, 35]. CHWs are often uniquely placed to attract resources for PHC initiatives from private individuals, corporations, foundations, bilateral and multilateral institutions [17, 18, 34, 35]. However we found that there was a mix of public, private, NGO, and traditional providers operating with variable population coverage and quality of services against BU [17, 18, 34, 35]. Advocating on behalf of PHC programmes that involve CHWs was frequently done by individual NGOs or groups of NGOs such as the *FAIRMED Foundation, ANESVAD, “Médecins Sans Frontières”, “Fondation Nipponne”, Raoul Foullereau, MAP International, and American Leprosy Missions* [17–19, 22, 25, 34, 35].

### Impact of CHWs

Three studies assessed the impact of CHWs activities over 12 months [19, 20, 30]. The studies demonstrated varying impacts of CHWs programmes on BU, ranging from a 67% [30] to 95% referring cases to the health centre [20]. CHWs identified and referred suspected cases of BU, of which 91% were confirmed cases. Most CHWs (78%) identified at least one suspected case of BU [20]. Two authors [19, 30] in Ivory Coast found that 67% and 65% of the cases referred by CHWs were confirmed by the medical staff. As concerns the proportion of pre-ulcerative forms of disease referred by CHWs, Vouking et al. [20] reported 85% in Cameroun and Ake et al. [19] found 38% in Ivory Coast.

The most important qualification of CHWs is implicit within the job title; the individual must be from the community that he or she will serve [20, 22, 23, 35]. The cultural, political and social contexts of the programme area influence the recruitment methods that are used and the quality criteria for CHWs. Overall, they are those that are most acceptable to the community [14, 32, 36]. In all the included studies, the CHWs were indeed recruited from the community [20–25].The training of CHWs is an important step in the fight against this disease [1, 29, 36, 37]. There is a very great need to train a large number of CHWs in order to improve the detection and management of cases [1, 29, 36]. While a large number of articles discuss or at least mention the training of CHWs, not surprisingly, the length, depth, organization of, responsibility for and approaches to training vary greatly across programmes [3, 29, 36]. When it was reported, the training approaches varied greatly between studies [20–27]. The terms used included: courses, classes, seminars, workshops, discussion groups, practical training, and in-class practice [20–22, 24, 26, 27].

One of the major obstacles to the control of BU is inadequate and poorly- equipped health facilities in the affected areas [6, 28, 29]. In line with the Yamoussoukro Declaration on BU [6], which called for the development of health systems so that effective treatment can reach all those affected, the actions of several NGOs are contributing to visible improvements in health facilities and the delivery of health services in the affected areas [3, 6, 28]. The crucial integration of CHWs programmes within the existing formal health systems and other programmes in the community is often initiated by NGOs [6, 37]. Unfortunately, those personnel who work closely with CHWs, in BU activities are not involved in the development and supervision of CHWs and therefore lend little support [29, 32, 36, 38, 39]. Strengthening the management capacity of district health teams to focus limited resources on priority problems can be done effectively in low-income settings, as shown by a BU programme that has placed powerful, but easy to use, decision-making methods, in the hands of local decision makers [40]. If CHWs are not trained, supported and supervised regularly on BU, the benefits of their work may be lost; therefore more efforts should be expended on the training of CHWs [12, 32] and the health personnel who work with them.

Our findings show the impact of health education and community surveillance strategies in BU control [20, 22, 41, 42]. This outcome could be explained by a good knowledge of the disease, and a good referral system in the endemic areas [20, 27, 42]. Early detection could reduce the cost of treatment and length of hospitalization as simple cases are treated within the health area [12, 20, 41].

Only three studies assessed the impact of CHWs activities over 12 months, showing impressive referral rates [19, 20, 30]. A recent review found that there is very little evidence on the effectiveness of CHWs in low-income countries [43, 44]. We found that their impact varied greatly. Thus, a key consideration for the design, implementation, and ongoing management of programmes for CHWs is how high-quality performance by CHWs will be achieved and maintained [45, 46]. Implementing CHWs programmes may require a little more than evidence of impact [44]. Factors such as feasibility and acceptability will vary by context and lead to different health and social outcomes [14, 43, 45].

### Study limitations

This review has several potential limitations. Firstly, it is possible that some published and unpublished CHWs evaluations were not identified through the search strategies used. However, considerable effort was made to identify additional studies by contacting the authors of included studies and scanning the reference lists of identified literature. Secondly, our definition of CHWs may have excluded some untrained lay health workers. Thirdly, the lack of uniformity in the study designs and reports rendered it impossible to make a comparative assessment of their quality. Finally, only three studies reported on the impact of CHWs, largely undermining our attempts to provide a detailed description of CHW impact in the control of BU.

## Conclusion

Evidence from this review suggests that CHWs programmes can have a considerable impact on the control of BU in sub-Saharan Africa. This may be related to high levels of knowledge of the disease by the CHWs, the early detection and a good referral system in the endemic areas. Facilitative supervision and availability of infrastructural support are critical issues for programme success; yet they are usually overlooked. Further studies are needed to assess the quality of care provided by CHWs for the management of BU.
